# Autotransplantation of a Tooth Cryopreserved Over 11 Years Using a Programmed Freezer With a Magnetic Field: A Case Report

**DOI:** 10.7759/cureus.52138

**Published:** 2024-01-11

**Authors:** Yuji Haneda, Saiji Shimoe, Toshitsugu Kawata, Masato Kaku

**Affiliations:** 1 Dentistry, Yutori Dental Clinic, Tokyo, JPN; 2 Department of Anatomy and Functional Restorations, Division of Oral Health Sciences, Hiroshima University Graduate School of Biomedical and Health Sciences, Hiroshima, JPN; 3 Orthodontics, Retired, University of Southern California, Los Angeles, USA

**Keywords:** periodontal ligament, magnetic field, cryopreservation, autotransplantation, missing tooth

## Abstract

This case report shows an autotransplantation of the lower right cryopreserved third molar into the extraction socket of the lower right first molar. Due to deep caries of the lower right first molar, the mesial root of this tooth was extracted. The patient asked to keep the distal root of the lower right first molar even if the root can survive only for a short period. So, a fixed partial denture supported by the lower right second premolar and the distal root of the lower right first molar was set. However, it was supposed that the distal root of the lower right first molar as an abutment tooth had a poor prognosis. Therefore, we also extracted the lower right third molar and cryopreserved to prepare autotransplantation if the lower right first molar has to be removed in the future. At first, the extracted third molar was frozen using a programmed freezer with a magnetic field named “Cells Alive System” (CAS) freezer, which was developed for tissue cryopreservation, and then, cryopreserved in the -150°C deep freezer. Eleven years later from the cryopreservation of the third molar, the lower right first molar showed root fracture. So, we extracted the lower right first molar and autotransplanted the cryopreserved third molar. Three years later, the autotransplanted tooth continued to be stable with healthy periodontium. The present case revealed that autotransplantation of a long-term cryopreserved tooth in a CAS freezer is a variable method to replace missing teeth.

## Introduction

Tooth autotransplantation is a useful method to replace missing teeth without defective dental restorations. Usually, an unnecessary third molar is selected as the donor tooth, because in most cases, the wisdom tooth does not affect dental occlusion. Because healthy periodontal ligament (PDL) tissues on autotransplanted teeth have osteoinduction ability, which can induce alveolar bone regeneration of the extraction socket [1,2], it is thought the prognosis of this treatment is good and reliable. However, patients often do not keep available wisdom teeth because it was previously extracted due to dental caries or pericoronitis. In order to avoid this problem, we established long-term teeth cryopreservation systems in a programmed freezer with a magnetic field named “Cells Alive System” (CAS) (ABI Corporation Ltd., Abiko, Japan). Previous reports showed that a CAS freezer with a 0.1-mT magnetic field could prevent ice crystal formation and achieve the greatest survival rate of PDL cells in vitro [3,4]. Also, in a clinical case, it was reported that cryopreserved upper second premolar by a CAS freezer was autotransplanted into the extracted socket of the lower left first molar after orthodontic treatment, showing a successful result [5]. However, autotransplantation of long-term cryopreserved teeth by a CAS freezer has not yet been reported. This case report shows a third molar autotransplantation into an extracted first molar space after an 11-year-cryopreservation in a CAS freezer.

## Case presentation

Pretreatment evaluation

**Figure 1:**
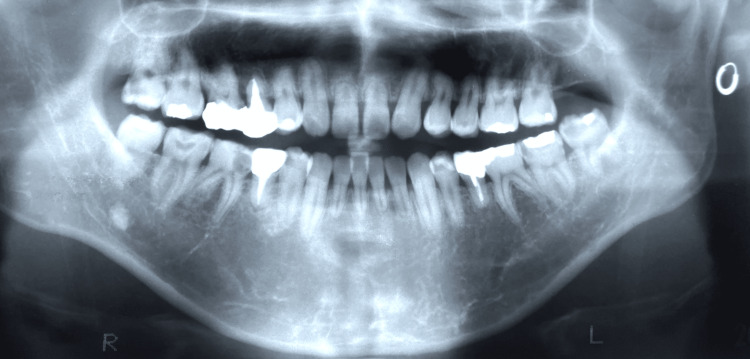
Pretreatment panoramic radiograph

The patient was a 28-year-old male with the chief complaint of pain in the lower right back tooth. The pre-treatment panoramic radiograph demonstrated deep caries on the lower right first molar. The mesial tooth crown almost collapsed and the caries reached the pulp tissue through enamel and dentine (Figure 1). 

The treatment started with treating the lower right first molar which was the main complaint. The treatment plan for the lower right first molar was done considering the degree of the mesial caries cavity which was too large; it was decided to extract the mesial root of the lower right first molar. The treatment plan was as follows: 1. Extraction of the mesial root of the lower right first molar; 2. Root canal treatment of the distal root of the lower right first molar; 3. Set a fixed partial denture on the lower right second premolar and the distal root of the first molar; 4. Extraction of the lower right third molar and cryopreservation for autotransplantation in the future.

Treatment progress

**Figure 2:**
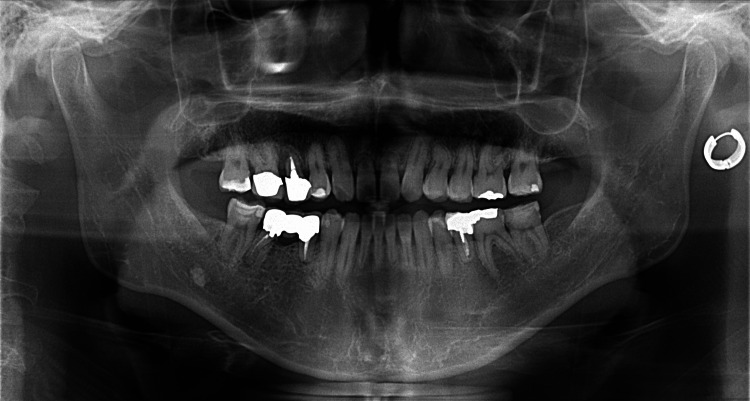
Panoramic radiograph during the treatment Panoramic radiograph after setting a fixed partial denture on the lower right second premolar and the distal root of the first molar.

Under local anesthesia, the mesial root of the lower right first molar was extracted. After root canal treatment of the distal root of the lower right first molar, a fixed partial denture was set on the lower right second premolar and the distal root of the first molar (Figure 2).

Then, the lower right third molar was extracted and cryopreserved, because the lower right first molar had a poor prognosis. The extracted third molar was put into the Teeth Keeper NEO immediately (Neo Dental International Inc., Federal way, USA), and transferred to Hiroshima University Hospital. Then, the teeth were transferred into 5 ml preservation media (Bambanker 2, Lymphotec, Tokyo, Japan) that contained 10% dimethyl sulfoxide (Me_2_SO) in a tempered hard-glass vial (SV-10, Nichiden-Rika Glass Co., Ltd, Kobe, Japan). A CAS-programmed freezer with a 0.1-mT magnetic field (ABI Corporation Ltd., Abiko, Japan) was used for initial freezing of the tooth from -5°C to -30°C at -0.5°C/min according to our previous study [3-6]. Then, the tooth was cryopreserved in a deep freezer at -150°C (MDF-1156ATN, PHC Holdings Co., Tokyo, Japan). 

Treatment results

**Figure 3:**
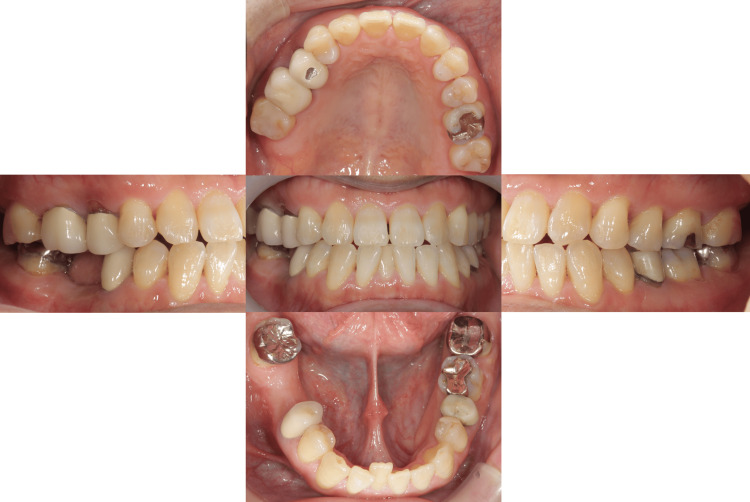
Intraoral photographs eleven years after setting the fixed partial denture The distal root of the lower right first molar was extracted.

**Figure 4:**
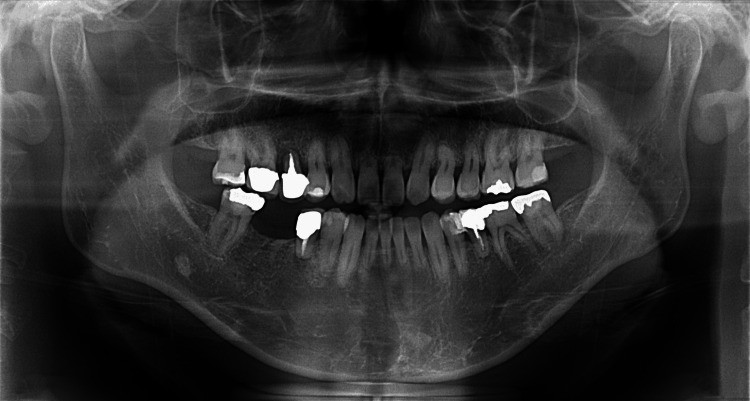
Panoramic radiograph after extraction of the distal root of the lower right first molar

Eleven years after setting the fixed partial denture, the patient had percussion pain on the lower right first molar. The distal root fracture and discharge of pus was observed on the lower right first molar. So, we decided to extract the distal root of the lower right first molar (Figures 3, 4).

Autotransplantation of the cryopreserved tooth

**Figure 5:**
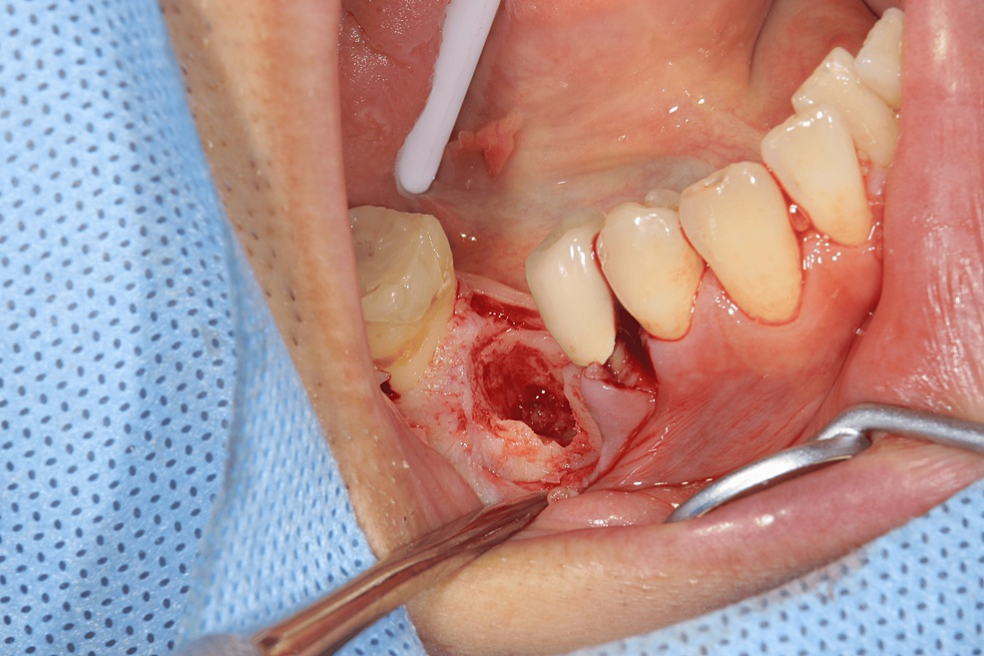
Intraoral photographs of the socket preparation for autotransplantation

**Figure 6:**
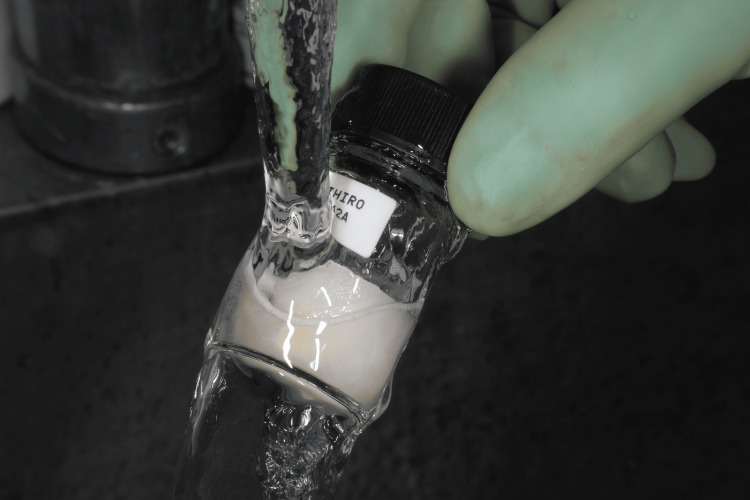
Thawing the cryopreserved tooth by a 37°C tapped water

**Figure 7:**
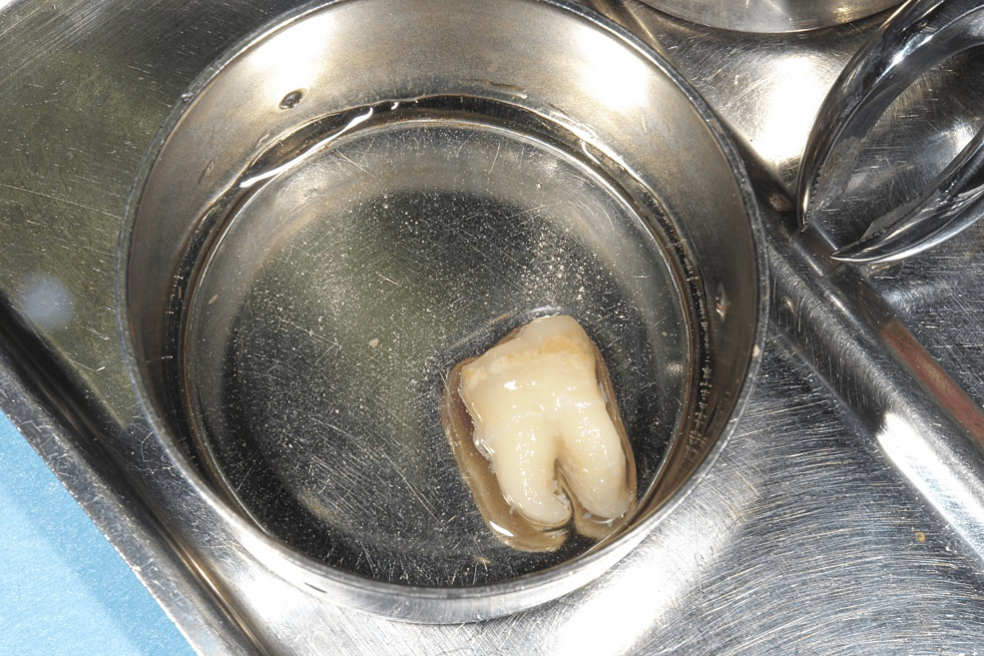
The cryopreserved third molar just before autotransplantation

**Figure 8:**
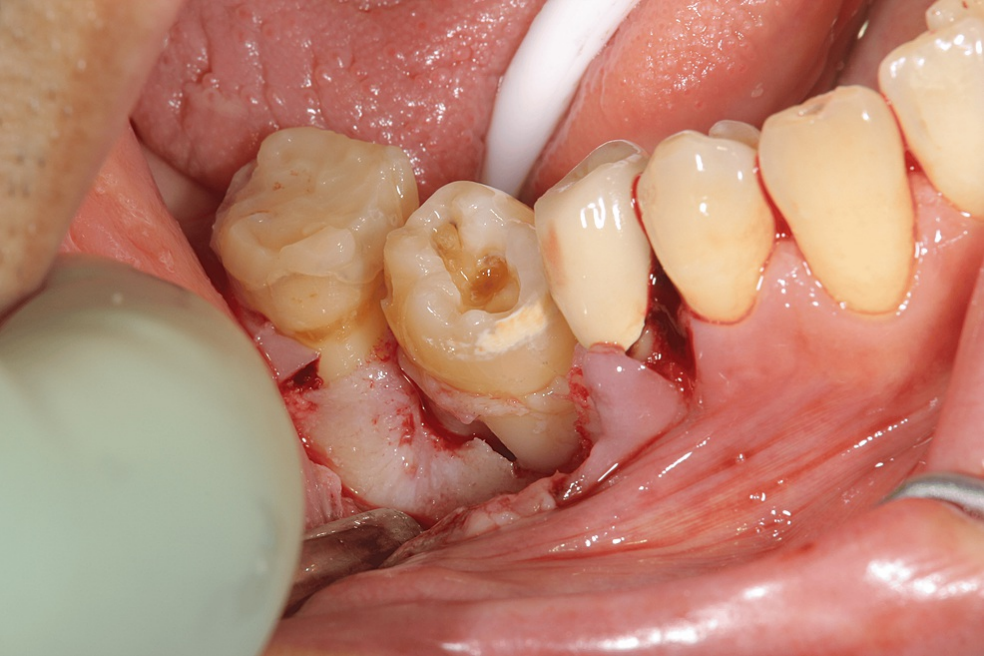
Immediately after autotransplantation of cryopreserved tooth

After the removal of the fixed partial denture and extraction of the lower right first molar, the socket preparation for autotransplantation was performed (Figure 5) and the cryopreserved third molar was autotransplanted into the sockets immediately after thawing by a 37°C tapped water and the sutured with a nylon thread (Figures 6-8).

**Figure 9:**
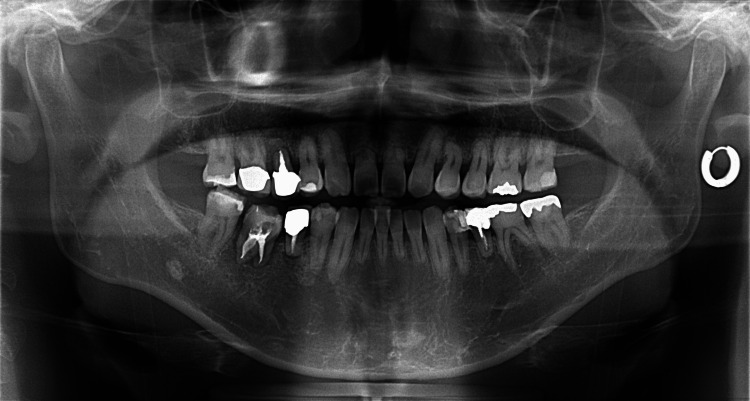
Panoramic radiograph after root canal treatment

Three weeks after autotransplantation, endodontic treatment and the root canal filling with gutta-percha point and sealer was finished (Figure 9).

**Figure 10:**
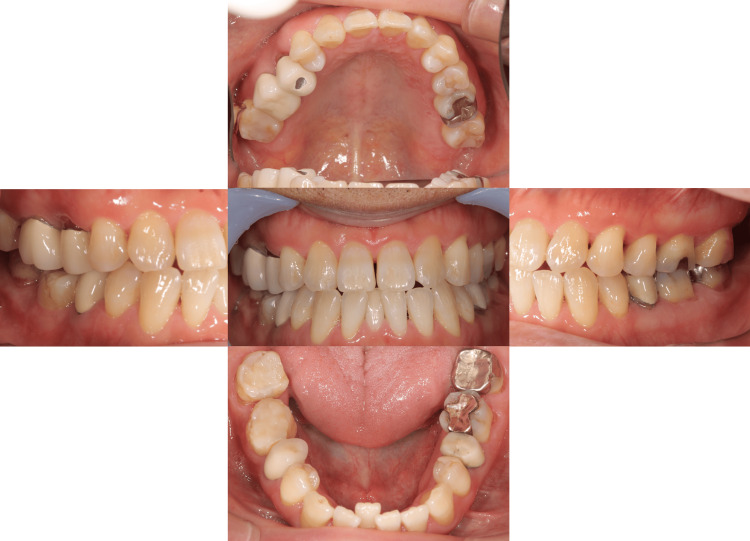
Intraoral photographs of the autotransplanted tooth The tooth crown was filled with composite resin.

Then, the transplanted tooth crown was filled with composite resin (Figure 10).

**Figure 11:**
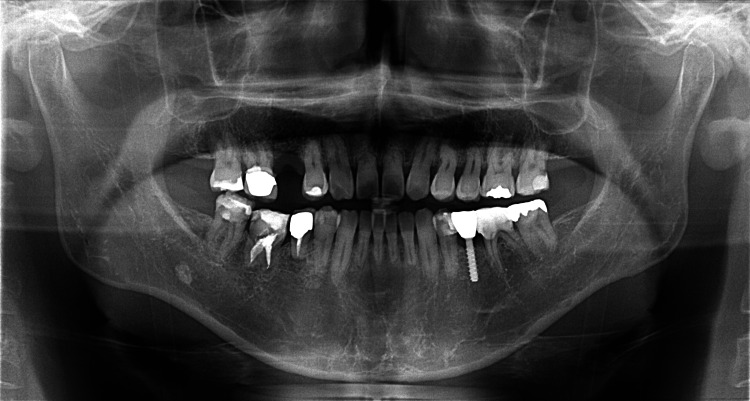
Panoramic radiograph three years after autotransplantation

Bone regeneration and physiological tooth mobility were observed, and inflammatory root resorption was not observed even three years after autotransplantation (Figure 11).

## Discussion

Teeth autotransplantation is a reliable treatment for the replacement of missing teeth. Long-term prognosis (17 to 41 years posttreatment) of teeth autotransplantation is also reported in the literature and exhibited that the survival rate was around 90% [7]. However, autotransplantation cannot be applied when suitable donor teeth are already extracted. So, many basic [8-10] and clinical [11-15] investigations of teeth cryopreservation have been performed long ago. For autotransplantation of cryopreserved tooth, the key to success factor is the survival of PDL cells, because tooth-bone ankylosis and root resorption can occur due to injured cryopreserved PDL cells by ice crystal formation. In order to solve this problem, a CAS-programmed freezer with a magnetic field has been developed. A magnetic field produced by a CAS freezer vibrates cells and water molecules, which can prevent the formation of ice crystals inside cells. It was shown that a 0.1-mT magnetic field, a 15-min hold-time, and a plunging temperature of -30°C led to the greatest survival and viability rate of PDL cells obtained from a one-year cryopreserved tooth. Any destruction of one-year cryopreserved PDL tissues by a CAS freezer was not found in the histological observations and transmission electron microscopic images, although critical cell injury was detected in tissues cryopreserved without a magnetic field [3]. Abedini et al. demonstrated that the PDL cells from a five-year cryopreserved tooth with a magnetic field could proliferate as much as that from an immediately extracted tooth [4]. Kamada et al. revealed that progressive root resorption was not seen in the teeth replanted after cryopreservation by a CAS freezer using a rat incisor replantation mode [6]. In the previous case study, bone regeneration with healthy periodontal ligament were observed after autotransplantation of a six-year cryopreserved tooth by a CAS freezer [5]. Furthermore, it was reported that a CAS freezer is useful for cryopreservation of human induced pluripotent stem cell-derived neural stem/progenitor cells. Cell proliferation and differentiation after thawing were significantly increased by a CAS freezer [16]. Kojima et al. showed that a CAS freezer can be available for high survival and proliferation rates of mesenchymal stem cells (MSCs) and can keep the ability of both adipogenic and osteogenic differentiation [17]. It was also reported that there were no differences of dental pulp stem cells in morphology, expression of stem cell markers, osteogenic and adipogenic differentiation between non-cryopreserved teeth and cryopreserved group by a CAS freezer [18]. Hashimoto et al. demonstrated that ice crystal formation was prevented in rat sciatic nerves that were freeze-thawed in a magnetic field [19]. Moreover, it was reported that a magnetic field suppressed the ice crystal damage of the tissues of tuna blocks, providing high-quality frozen foods [20].

Also, in the present case, inflammatory root resorption and replacement resorption were not seen after autotransplantation of an 11-year cryopreserved tooth by a CAS freezer and the tooth continued to be stable three years after autotransplantation. This case report clearly demonstrated that a CAS freezer is useful for long-term tooth cryopreservation and later autotransplantation of unnecessary wisdom teeth and extracted teeth for orthodontic treatment for recovering occlusal function.

## Conclusions

This case report demonstrates an autotransplantation of the lower right cryopreserved third molar into the extraction socket of the lower right first molar. The lower right third molar was cryopreserved using a CAS freezer for 11 years before autotransplantation. The autotransplanted tooth exhibited stable and healthy periodontal tissue three years after the autotransplantation. Thus, a CAS freezer is available for long-term tooth cryopreservation and later autotransplantation.
